# Effectiveness of Stocking Density Reduction on Mitigating Lameness in a Charolais Finishing Beef Cattle Farm

**DOI:** 10.3390/ani10071147

**Published:** 2020-07-07

**Authors:** Martina Cortese, Marta Brščić, Nicola Ughelini, Igino Andrighetto, Barbara Contiero, Giorgio Marchesini

**Affiliations:** Dipartimento di Medicina Animale, Produzioni e Salute, Università degli Studi di Padova, 35020 Legnaro, Italy; marta.brscic@unipd.it (M.B.); nicola.ughelini@gmail.com (N.U.); igino.andrighetto@unipd.it (I.A.); barbara.contiero@unipd.it (B.C.); giorgio.marchesini@unipd.it (G.M.)

**Keywords:** beef cattle, animal welfare, space allowance, average daily gain, health

## Abstract

**Simple Summary:**

In finishing beef cattle farms, the limited space allowance and the fully slatted floor system could negatively affect animals’ performance, health and welfare. A larger individual space was provided as a strategy to mitigate lameness problems in a commercial farm. A larger space allowance may contain the spread of pathogens, leading to a reduction in the incidence of lameness and infectious diseases, and may decrease the risk of animals stepping into each other causing claw and leg injuries. Increasing the space allowance did not affect animal performance, whereas it had a positive implication through a significant reduction in the number of treatments of lame and sick animals.

**Abstract:**

This study aimed at assessing whether a reduction in stocking density (SKD) would mitigate lameness and positively affect the performance and health of Charolais bulls in an Italian commercial farm. Bulls were distributed in groups of 10 or 8 animals/pen for high (HD) or low density (LD) corresponding to an individual space of 3.5 or 4.7 m^2^, respectively. Bulls were fitted with collars that measured rumination time and activity. Three 8-h observational sessions were conducted to record behaviors. Data about health conditions were collected daily. No differences were found in the animals’ performance. However, performance results might have been impaired by the culling rate experienced during the trial, which prevented from keeping a consistent SKD. Behaviors did not differ between groups, except for rumination time, which was higher for LD bulls during the third observation (*p* < 0.05). However, rumination time, recorded by collars, did not vary among treatments. There were no differences in the percentage of sick or lame bulls, but the percentage of animals treated repeatedly due to relapse was higher for the HD group (*p* < 0.05). It was concluded that a larger space allowance could improve the health of bulls kept on fully slatted floors.

## 1. Introduction

Beef cattle production is particularly relevant in Italy, which is the fourth main contributor for European production [1]. The main production system is based on fattening of young beef cattle breeds and about 45% of them are imported from abroad (mainly from France, Ireland and Eastern European countries) [2]. Charolais is one of the most imported French purebreds finished in Italy. This breed is generally slaughtered at a heavy body weight (BW) (over 700 kg) and during the finishing phase, bulls are kept in indoor slatted floor systems. This flooring system is the most frequently adopted system in Europe since it requires the smallest space allowance [3], less management labor and no need for litter renewal [4]; thus, its slipperiness may lead to foot and leg injuries. The use of slatted concrete floor has been demonstrated to increase the risk of culling due to severe lameness by about threefold [5]. Several other factors are known to affect beef cattle welfare and performance on farms, and among these, space allowance can have a major impact on beef cattle welfare, behavior and health [6,7,8]. Limited space allowance has been pointed out as a relevant risk factor for beef cattle health by the European Food Safety Authority (EFSA) Panel on Animal Health and Welfare [9]. Specifically, it could constrain animals to abnormal lying down and standing up movements or cause animals to step on lying pen-mates, contributing to a further increase of the risk of lameness [5,6]. Inadequate floor space may reduce resting time, increase the episodes of disturbance of lying bulls and increase the frequency of aggressive behaviors [10]. The Scientific Committee on Animal Health and Animal Welfare (SCAHAW) [3] addressed that a minimum of 3.0 m^2^ should be guaranteed for animals expected to reach 500 kg, plus 0.5 m^2^ for each 100 kg over 500 kg. However, there are no current European regulations that contextualize standards regarding space allowance for fattening bulls. 

A larger space allowance may lead to a higher average daily gain (ADG) and feed conversion ratio (FCR). In fact, stress affects gonads, resulting in lower hormonal secretion, which may affect performance [11]. Moreover, stress may decrease dry matter intake (DMI) and influence gastrointestinal function, decreasing nutrient absorption and digestibility [8]. According to Ingvartsen et al., a space allowance of less than 4.7 m^2^ per bull weighing 250–500 kg was found to reduce ADG up to 300 g, owing to the drop in feed intake. 

Genetic selection over the years has focused on hypermuscularity traits which, at the same time, might exert a negative effect on immunological responsiveness, making bulls more vulnerable to stressors and pathogens [12,13]. Furthermore, crowding and stressful conditions facilitate a higher spread of pathogens. The transmission of respiratory diseases is favored by nose-to-nose contacts or environmental and fomite exposures [14]. Limiting animal contact with pathogens plays an important role in declining the transmission of diseases [15]. 

We hypothesized that a larger space allowance could reduce the prevalence of lame bulls, improve health and positively affect animal performance in commercial farms characterized by a high incidence of lameness. This condition may be obtained by decreasing the number of animals raised per pen. Thus, the aim of the study was to test the effect of a reduction in stocking density on Charolais finishing beef cattle kept on fully slatted floor in a commercial farm with a high prevalence of lameness, in the perspective of reducing claw and leg problems and improving animal health and production efficiency.

## 2. Materials and Methods

### 2.1. Animals, and Feeding and Experimental Design

The trial was carried out in a commercial beef cattle farm in the Rovigo province, in northeastern Italy, from July to October 2018, not applying any procedure that goes beyond the regular commercial production practice, as approved by OPBA (Organismo Preposto al Benessere Animale—Animal Welfare at ISS committee). The farm happened to have a high prevalence (on average 15%) of lame bulls during the finishing phase in the previous years. Lameness was caused, among other factors, by digital dermatitis due to *Treponema* species. According to the farm biosecurity procedure set by the farm veterinarian, each pen was sanitized before the arrival of the successive occupants with the use of limestone and left idle for a short period of time. The study involved a group of Charolais bulls (*n* = 108 animals), which were purchased from the same cattle collection center in France and selected to be homogenous in weight (424 ± 30 kg), age (466 ± 55 days) and physical conformation. The minimum sample size for each group was reckoned taking into account a power of the test of 90%, a standard deviation of the ADG of 0.2 kg/d and a significant difference in ADG of 0.133 kg/d.

After spending 60 days for their backgrounding and transition period in a loose housing facility with straw bedding, the animals were moved into 12 contiguous, concrete, fully slatted pens (5 m wide and 7 m deep) in a roofed facility with full walls on three sides, for their growing (70 d) and finishing (61 d) phases.

Animals were divided into two groups, homogenous in weight and housed according to two levels of stocking density (6 pens each in alternative order). The two levels of stocking density were 10 and 8 animals per pen corresponding to 3.50 m^2^/animal for the high-density group (HD) and 4.37 m^2^/animal for the low-density group (LD), respectively. The HD condition was the one routinely adopted by the farmer, whereas the LD condition was the one suggested by the research group as a compromise between animals’ and farmer’s needs. The stocking density within the pens changed over time because of severe culling. Therefore, to analyze the effect of density, we kept maintaining the LD category for pens where the average number of animals per day, within each period, was equal or lower than 8.0, whereas this value was higher in the HD category (Table 1). The average number of animals per pen during each period was given by the following formula:

(1) $$\text{Average number of animals per pen}=\frac{\sum_{1}^{i}n}{i}$$

where *n* is the number of animals present per day in a pen and *i* is the number of days of the corresponding period.

### 2.2. Growth Performance, Ration, Digestibility and Diet Particle Length Distribution

Bulls were fed the same total mixed ration (TMR). The ingredients and chemical composition of the diet are reported in Table 2. 

To meet animals’ requirements, diet adjustments were made when bulls were moved from the growing phase (P1) into the finishing phase (P2). The day before the beginning of the trial, bulls were fitted with SCR collars (HRLDn Tag; SCR Engineers) to measure their daily activity and rumination.

Overall, the trial lasted 131 days. All bulls were weighed in the morning before feed distribution at the outset (day 1), at day 70, between the growing and finishing phases, and at the end of the trial, to calculate individual ADG. The DMI was reckoned for each pen by the difference between the amounts of TMR given in the morning (T0) and its residuals after 24 h. Pen feed conversion rate (FCR) was calculated by dividing DMI by ADG. For both diets, TMR and 24-h leftover samples were collected each week, whereas 2 fecal samples per pen were gathered once per period. The particle length distribution of TMR samples was obtained by using the updated version of the Penn State Particle Separator [16] that is characterized by 3 sieves of 19, 8 and 4 mm and a bottom pan. Feeds and feces were dried at 60 °C for 48 h and ground to pass a 1-mm screen and analyzed for dry matter (DM), ash, crude protein (CP), neutral detergent fiber (NDF), acid detergent lignin (ADL) and starch following the procedures reported in the literature [17].

Total tract digestibility was estimated using lignin as an internal marker as described by McDonald et al. [18] and Schäfers et al. [19] to calculate the digestibility of DM (DMD), CP (CPD), NDF (NDFD) and starch (Starch_D) as follows:

(2) $$\mathrm{DMD}=\frac{\text{marker in faeces}\;\left(g/kg\;DM\right)-\text{marker in feed}\;\left(g/kg\;DM\right)}{\text{marker in faeces}\;\left(g/kg\;DM\right)}$$

(3) $$\mathrm{ND}=1-\frac{\text{marker in diet}\;\left(g/kg\;DM\right)\times N\;\text{in faeces}\;\left(g/kg\;DM\right)}{\text{marker in faeces}\;\left(g/kg\;DM\right)\times N\;\text{in diet}\;\left(g/kg\;DM\right)}$$

where N is the nutrient and ND is the nutrient digestibility.

### 2.3. Animal Behavior

Behavioral observations were carried out three times during the experimental period, in 8-h sessions starting right after feed distribution at 09:30. Animals were observed by four trained assessors standing outside the pens in the feeding alley. Each assessor observed animals in 6 contiguous pens for 2 h, alternatively monitoring 3 HD and 3 LD pens (2-h observation, 2-h pause). To reduce bias due to the observer effect, observers rotated every 2 h and changed position at each 2-h interval. Behavioral assessments of postures (standing/lying) and continuous different activities (eating, ruminating, being inactive, resting or involved in other activities) of bulls in each pen were performed using the scan sampling technique [20] with a 5-min interval between two consecutive scans (see supplementary file). Mounting, chasing, head/butt displacement and drinking were noted as events as they occurred (1 = occurrence) at pen level, following the behavior sampling procedure proposed by Martin and Bateson [20].

### 2.4. Monitoring, Clinical Assessments and Treatments

The individual health condition of the bulls was checked daily by the stock people as required by the legislation in force on farm animal protection and weekly by an experienced veterinarian throughout the trial. The animals were visually inspected from the feeding alley and the occurrence of any symptom was recorded. Health check recordings were performed according to the Welfare Quality^®^ assessment protocol for cattle [21] and special attention was given to lameness, defined by Van Hertem et al. [22] as an alteration of gait caused by leg or hoof injuries or disorders. The number of treatments and the number of early culled animals due to fatal or traumatic events or lameness were recorded as well.

To measure the rumination time and the level of activity of individual animals, SCR collars were used. These collars are equipped with an internal accelerometer which records the movements of the head and sends the data to a receiver connected to a computer at 2-h intervals. The computer software (Heatime Pro System/HRLDn Tag; SCR Engineers, Netanya, Israel) differentiates the type of movements and reckons the rumination time and the level of activity, ranging from 0 to 253 bits (binary digit), per interval [23]. It gives an indication of how much the animal has moved, without discriminating the kind of action. Data were summarized on a daily basis. The system was validated for both rumination and activity by Schirmann et al. [24,25] and tested for the detection of sick beef cattle by Marchesini et al. [26] through the use of average daily rumination time and activity and the dishomogeneity indices for activity (DA) and rumination (DR), which were calculated according to Marchesini et al. [27].

### 2.5. Statistical Analysis and Calculations

A statistical analysis was carried out using the SAS software (release 9.4; SAS Institute Inc., Cary, NC, USA). All data were first tested for normal distribution using the Shapiro–Wilk test (a value higher than 0.9 meant that data had a normal distribution). Data on diet particle length distribution were submitted to an ANOVA model using period as the fixed effect. Data on DMI, ADG, FCR, digestibility, rumination and activity were analyzed through a mixed ANOVA model using the pen within stocking density as the random effect and period, stocking density and their interaction as fixed effects. Bonferroni correction was run for post-hoc pairwise comparisons between factor levels. Assumptions of the linear model on the residuals were graphically tested.

Data on the number of treatments per animal were submitted to the non-parametric Kruskal–Wallis test to evaluate the effects of stocking density, whereas data on the number of animals treated or culled, expressed as percentages, were analyzed through a Z test and the culling rate was analyzed through an Fisher’s exact test.

Behavioral data gathered using the scan sampling technique were transformed into relative frequencies of events per pen per hour and were processed using a mixed model that considered the main effects of observation session day, stocking density and their interaction using the Bonferroni adjustment. Data regarding events were transformed into number of events per bull in the 8-h observation session and submitted to non-parametric statistics using the Kruskal–Wallis test.

## 3. Results

As previously anticipated, SKD changed throughout the trial, as reported in Table 1.

Diet ingredients and chemical composition are listed in Table 2.

During the experiment, 35 animals needed to be treated for different kinds of problems. Specifically, 26 animals suffered from lameness, 4 from respiratory affections, 2 from leg lesions, 2 from urogenital problems and 1 from diarrhea. Once treated and recovered, 11 out of the 26 lame bulls and 1 out of the 4 bulls which had suffered from respiratory problems experienced a relapse, needing a second pharmacological treatment. Overall, 16 bulls, of which 4 belonged to LD and 12 to HD, were culled early due to severe lameness. The effects of SKD on health status are described in Table 3. No differences were observed for the percentage of treated animals and early culled bulls, or for the average number of animals treated per pen. In regard to relapses, less repeated treatments were recorded per each sick (*p* = 0.011) and lame animal (*p* = 0.032) in LD pens.

The particle length distribution is reported in Table 4. Period 2 was characterized by a higher (*p* < 0.001) content of medium (8–19 mm) particles and a lower (*p* < 0.001) content of short (4–8 mm) particles.

As reported in Table 5, animals had similar BWs in both SKD groups at the beginning of the study. There was no significant effect of either the diet or the stocking density on final BW, ADG, DMI, FCR and digestibility of nutrients. Period affected many variables: bulls in the second period showed higher initial and final BWs (*p* < 0.001) and DMI (*p* < 0.001) and lower digestibility of both DM (*p* = 0.014) and NDF (*p* = 0.003). Since the interaction period × stocking density was not significant, it was not reported in the table.

Results obtained from SCR collar recordings of rumination and activity are shown in Table 6. Daily activity was higher in LD animals compared with HD (*p* = 0.015), whereas daily rumination, DA and DR did not differ between different diets and SKD. With regard to the effect of period, significance was approached for daily rumination, DA and DR, which were all higher in P1 than in P2 (*p* < 0.001). As for previously reported data, the interaction of period × SKD did not approach significance, thus it was omitted.

Regarding the effect of the interaction between SKD and observation session, reported in Table 7, only rumination (*p* = 0.024) was affected. No differences in social interactions like mounting, chasing and head/butt displacement were found between the two SKDs.

## 4. Discussion

Overall, in this trial, although the stocking density did not significantly affect the prevalence of sick or lame animals, it led to a significant change in the number of treatments necessary to an animal to fully recover from lameness or other diseases. Looking at the average number of treatments per sick or lame animal, it can be appreciated that, among the debilitated animals, bulls raised in HD conditions required a higher number of pharmacological interventions to recover or that they experienced more relapses. Indeed, a smaller space allowance favors contacts between animals and pathogen transmission and increases animal stress, thus weakening the immune system [15]. Based on our findings, it could be deduced that there are more chances of a complete recovery after the onset of lameness or other pathologies with a larger space allowance. Moreover, animals housed on fully slatted concrete floors have a higher risk of slipping or stepping on a lying pen-mate, increasing the risk of lameness. The risk of traumatic injuries may be reduced by providing a larger space allowance, as reported by other authors [5].

As regards the TMR, the distribution of particle length was partitioned similarly to what was reported in previous studies [27,28], where similar rations were fed. As animals entered into P2, rations were changed to meet growth requirements at the finishing phase. Specifically, NDF content was reduced to 28.7%, whereas starch was increased up to 34.2%. This modification can justify the slight difference in particle size distribution found between periods.

Against our expectation, stocking density did not affect bulls’ performance. We expected that animals raised with a larger space allowance would have an increase in ADG. In fact, previous studies have shown that animals with a small space allowance have a shorter lying time which leads to a reduced daily gain [29]. Marquis et al. [30] reported an increase of 300 g of ADG when space allowance was increased from 1.5 to 2.0 m^2^ in a study involving 237 steers of 450 kg of weight. The lack of association between average daily gain and space allowance found in our experiment could partially be explained by the culling rate we recorded throughout the trial. As a matter of fact, animals started with 3.5 and 4.37 m^2^ of space allowance for HD and LD, respectively. Nevertheless, they ended up having on average 3.68 m^2^ in HD and 4.42 m^2^ in LD during P1, and 4.07 m^2^ in HD and 4.62 m^2^ in LD during P2, and this might be seen as the main shortcoming of our study. It implied that the difference between the two levels of space allowance passed from 0.87 m^2^ per head at the beginning of the trial to 0.55 m^2^ in the second period, which likely was not great enough to affect ADG.

As animals grow, DMI increases proportionally to their metabolic body weight [31]; thus, DMI increased from P1 to P2. The decrease in NDF content in the diets from P1 to P2 determined an increase of the passage rate of the ingesta. For this reason, DMD and NDFD were higher in P1 compared with P2. In fact, at a higher DMI, it seems likely that a faster rate of passage of ingesta through the digestive tract may cause a decrease of digestibility of nutrients [32].

With respect to SCR collars recordings, activity recorded for LD bulls was higher compared with HD bulls, meaning that when bulls had more space, they were more likely to move. From P1 to P2, we observed a decrease in daily rumination (min) and DR, which meant that there was less variation in rumination between one day and another in the second part of the experiment [26]. This was explained by the drop of peNDF in P2, which is known to be an important stimulus to rumination [16,33].

The only behavior affected by treatment was ruminating activity which, during the third observation, was higher for LD compared with HD. This was in line with what had been found by Fisher et al. [13]; in an experiment involving finishing heifers kept at 1.5 m^2^, 2.0 m^2^, 2.5 m^2^ and 3 m^2^, they recorded a reduced level of rumination for heifers housed in the 1.5 m^2^ space allowance. They attributed the result to a lower lying times of these animals. However, we did not record any changes for the latter. Thus, these different conclusions may be related to variations in the study designs other than space area like the different sex and breed. Moreover, if we look at rumination data recorded by SCR collars, no difference among groups can be appreciated. The reason for the difference between groups noted at the third observation session could be linked to a possible compensation that occurred during the rest of the day. In fact, collars recorded rumination time on a 24-h basis, and any fluctuation during the 8-h observational session might have been compensated in the nighttime.

## 5. Conclusions

Increasing space allowance improved animals’ health status, decreasing the need of further treatments due to relapses for sick and lame bulls. However, it was of no benefit to bulls’ performance, but it must be considered that the planned space allowance could not be maintained, due to the culling rate experienced throughout the trial. Despite the fact that we cannot draw robust inferences, both due to the changes in stocking density and the limited number of animals, results of this study could spur beef cattle farmers to increase space allowance to improve animal health status and welfare, in high lameness prevalence conditions in particular. Further research is needed to address the importance of increasing space allowance and investigate which is the adequate space allowance for Charolais bulls housed in indoor facilities.

## Figures and Tables

**Table 1:** Changing of stocking density (number of animals per pen), related stocking density class (low density (LD) vs. high density (HD)) and individual space area per animal in pens over time (growing phase (P1) and finishing phase (P2)), due to early culling.

Pen	Initial SKD			Average SKD P1			Average SKD P2		
	*N*	m^2^/head	Class	*N*	m^2^/head	Class	*N*	m^2^/head	Class
Pen 1	8	4.37	LD	8	4.37	LD	8	4.37	LD
Pen 2	10	3.50	HD	9.5	3.68	HD	8.5	4.12	HD
Pen 3	8	4.37	LD	8	4.37	LD	8	4.37	LD
Pen 4	10	3.50	HD	9.5	3.68	HD	8.5	4.12	HD
Pen 5	8	4.37	LD	8	4.37	LD	7.5	4.66	LD
Pen 6	10	3.50	HD	10	3.50	HD	9.5	3.68	HD
Pen 7	8	4.37	LD	8	4.37	LD	7.5	4.66	LD
Pen 8	10	3.50	HD	10	3.50	HD	10	3.50	HD
Pen 9	8	4.37	LD	8	4.37	LD	7.5	4.66	LD
Pen 10	10	3.50	HD	9.5	3.68	HD	8	4.37	LD
Pen 11	8	4.37	LD	7.5	4.66	LD	7	5.00	LD
Pen 12	10	3.50	HD	8.5	4.12	HD	7	5.00	LD

SKD = stocking density; *N* = average number of animals per pen; LD = low density; HD = high density.

**Table 2:** Diet ingredients and chemical composition of total mixed rations (TMRs) of period 1 and period 2 (P1 and P2).

Items	Period	
	P1	P2
Ingredients (% of DM)		
Bran	17.3	15.0
Corn meal	24.9	34.8
Straw	6.52	5.67
Corn silage	20.5	17.8
Soybean meal	6.52	5.67
Pressed beet pulps	20.5	17.8
Protein, vit/min mix	3.68	3.20
Proximate composition (% of DM)		
DM	47.8 ± 2.42	47.3 ± 2.10
CP	13.7 ± 0.74	13.3 ± 0.88
NDF	35.2 ± 1.68	28.8 ± 1.42
peNDF	22.7 ± 0.89	18.6 ± 0.33
Starch	28. 9 ± 1.50	34.2 ± 1.67

Protein, vit/min mix = protein, vitamin and mineral mix; DM = dry matter; CP = crude protein; NDF = neutral detergent fiber; peNDF = physically effective neutral detergent fibre.

**Table 3:** Effect of stocking density (LD and HD) on the percentage of treated bulls and early culled bulls and the number of treatments per Charolais bull.

Health Data	SKD		*p*-Value
	LD	HD	
Treated bulls (%) ^1^	14.7	21.5	0.271
Treated bulls for lameness (%) ^1^	12.7	18.7	0.319
Early culled bulls (%) ^2^	8.33	20	0.108
Prevalence of total diseases (% per pen) ^3^	0.24	0.15	0.432
Prevalence of lameness (% per pen) ^3^	0.21	0.14	0.431
Number of treatments per sick animal (average per pen) ^3^	1.23	2.60	0.011
Number of treatments per lame animal (average per pen) ^3^	1.01	2.30	0.032

SKD = stocking density; LD = low density; HD = high density; ^1^ Estimated using a Z test; ^2^ Estimated using Fisher’s exact test; ^3^ Estimated using a non-parametric Kruskal–Wallis test.

**Table 4:** Particle length distribution of diet in periods P1 and P2.

Particle Length Distribution (%)	Period		SEM	*p*-Value
	P1	P2		
>19 mm	5.05	4.48	0.39	0.232
8–19 mm	16.5	21.8	0.81	<0.001
4–8 mm	44.1	39.9	0.84	<0.001
Bottom pan	34.3	33.8	0.97	0.654

SEM = standard error of mean.

**Table 5:** Effect of period (P1 and P2) and stocking density (LD and HD) on dry matter intake (DMI), average daily gain (ADG), feed conversion ratio (FCR) and digestibility of dry matter, crude protein, neutral detergent fiber and starch (DMD, CPD, NDFD and starch_D, respectively).

Items	Period		SKD		SEM	*p*-Value	
	P1	P2	LD	HD		Period	SKD
Initial BW	512	604	567	548	12.4	<0.001	0.256
Final BW	603	681	651	630	14.3	<0.001	0.326
ADG	1.33	1.25	1.32	1.24	0.110	0.418	0.466
DMI	9.88	10.6	10.1	10.6	0.31	<0.001	0.256
FCR	7.84	8.62	7.99	8.82	0.720	0.208	0.276
DMD	66.7	63.2	64.2	65.7	1.58	0.028	0.397
CPD	51.7	53.0	51.1	53.5	1.84	0.517	0.235
NDFD	53.6	48.1	50.4	51.3	1.85	0.007	0.640
Starch_D	97.2	97.4	97.2	97.4	0.55	0.647	0.651

SEM = standard error of mean; BW = body weight; SKD = stocking density; LD = low density; HD = high density.

**Table 6:** Effect of stocking density (SKD)and period (P1 and P2) on daily activity, daily rumination, index of dishomogeneity in activity (DA) and index of dishomogeneity in rumination (DR).

Items	Period		SKD		SEM	*p*-Value	
	P1	P2	LD	HD		Period	SKD
Daily activity (bit)	373	378	384	368	5.6	0.355	0.015
Daily rumination (min)	342	299	320	321	11.6	<0.001	0.936
DA	0.125	0.105	0.114	0.117	0.004	<0.001	0.523
DR	0.291	0.260	0.279	0.271	0.008	<0.001	0.417

SEM = standard error of mean; SKD = stocking density; LD = low density; HD = high density.

**Table 7:** Effect of stocking density on the bulls’ behavioral activities recorded during three 8-h observation sessions throughout the trial.

Behavioral Activity (% of bulls)	LD			HD			SEM	*p*-Value
	1st	2nd	3rd	1st	2nd	3rd		Obs × SKD
Lying	49.0	57.2	51.8	45.7	55.8	51.6	3.08	0.865
Eating	8.58	9.37	8.47	8.12	6.93	8.36	1.001	0.408
Ruminating	14.2 ^ab^	13.7 ^b^	16.5 ^a^	13.6 ^b^	15.2 ^ab^	11.3 ^b^	1.07	0.024
Exploring	1.13	1.39	1.44	0.91	1.11	0.63	0.195	0.369
Allogrooming	2.62	2.99	3.11	1.89	1.96	3.17	0.426	0.471
Self-grooming	2.57	3.49	2.92	1.80	3.50	2.60	0.437	0.557
Resting	13.8	28.1	18.1	13.8	25.6	15.7	2.04	0.653
Inactive	48.3	31.4	39.7	50.2	36.3	49.0	2.36	0.135
Others	9.75	10.53	9.63	9.70	9.26	10.30	0.720	0.256

SEM = standard error of mean; Obs = observation; SKD = stocking density; LD = low density; HD = high density; ^a, b^ Values with different superscript letters significantly differ (*p* < 0.05).

## Supplementary Materials

The following are available online at https://www.mdpi.com/2076-2615/10/7/1147/s1, Table S1: Ethogram of bulls.
