# Supplementary material for: Effectiveness of Stocking Density Reduction on Mitigating Lameness in a Charolais Finishing Beef Cattle Farm
Source: Animals (Basel). 2020 Jul 7;10(7):1147. doi: 10.3390/ani10071147 (PMC7401523; doi:10.3390/ani10071147)
Supplement: Supplementary file 1 [file animals-10-01147-s001.pdf]

Supplementary Materials

Effectiveness of Stocking Density Reduction on Mitigating Lameness in a Charolais Finishing Beef Cattle Farm

Martina Cortese, Marta Brščić, Nicola Ughelini, Igino Andrighetto, Barbara Contiero and Giorgio Marchesini

Table S1. Ethogram of bulls.

CATEGORY OF BEHAVIOUR	BEHAVIOUR	DEFINITION
Posture	Standing	Bull stands at three or four legs
	Lying	Bulls lies on side with four legs stretched, with 1, 2 or 3 legs stretched but close to the body, or lies on the sternum with four legs under its body
Behaviour		
General	Walking	Bull walks through the pen at a regular pace (not run/jump/frolic behaviour) performing 3 or more steps without stopping
	Exploring	Bull licks, nibbles, sniffs, sucks, or bites at the fence, wall, or other object not in a stereotypical way
	Inactive	Bull just looking ahead; no activity
	Other	Behaviour not included in the ethogram; behaviour of the bull is not visible for the observer
Feeding behaviour	Eating	Bull actively intakes the feed at the manger including chewing it in the manger space
	Ruminating	Bull performs chewing movements; it begins when the bull starts to chew a regurgitated bolus and it ends when the bolus is swallowed back.
	Drinking water	Bull drinks water out of the pressure bowl
Comfort and social behaviours	Resting	Bull lies, not moving or ruminating
	Self-grooming	Bull licks himself; including snout/head licking
	Allogrooming	Bull licks or nibbles a pen mate not in a stereotypical way
